# Persistence in Epidemic Metapopulations: Quantifying the Rescue Effects for Measles, Mumps, Rubella and Whooping Cough

**DOI:** 10.1371/journal.pone.0074696

**Published:** 2013-09-09

**Authors:** C. Jessica E. Metcalf, Katie Hampson, Andrew J. Tatem, Bryan T. Grenfell, Ottar N. Bjørnstad

**Affiliations:** 1 Department of Zoology, Oxford University, Oxford, Oxfordshire, United Kingdom; 2 Fogarty International Center; National Institute of Health, Bethesda, Maryland, United States of America; 3 Department of Ecology and Evolutionary Biology, Princeton University, Princeton, New Jersey, United States of America; 4 Institute of Biodiversity, Animal Health and Comparative Medicine, University of Glasgow, Glasgow, United Kingdom; 5 Department of Geography and Environment, University of Southampton, Southampton, Hampshire, United Kingdom; 6 Centre for Infectious Disease Dynamics, Pennsylvania State University, State College, Pennsylvania, United States of America; Melbourne School of Population Health, Australia

## Abstract

Metapopulation rescue effects are thought to be key to the persistence of many acute immunizing infections. Yet the enhancement of persistence through spatial coupling has not been previously quantified. Here we estimate the metapopulation rescue effects for four childhood infections using global WHO reported incidence data by comparing persistence on island countries *vs* all other countries, while controlling for key variables such as vaccine cover, birth rates and economic development. The relative risk of extinction on islands is significantly higher, and approximately double the risk of extinction in mainland countries. Furthermore, as may be expected, infections with longer infectious periods tend to have the strongest metapopulation rescue effects. Our results quantitate the notion that demography and local community size controls disease persistence.

## Introduction

Highly contagious pathogens can invade host populations fast and use the susceptible pool very efficiently. The consequence of this for immunizing infections such as measles, mumps, rubella and whooping cough, is that local chains of transmission can break and cause local extinction as the susceptible pool is depleted. It has long been recognized that these infections may depend on metapopulation dynamics whereby local persistence is impossible, but recolonization (‘rescue effects’) among spatially separate communities with asynchronous epidemics can ensure regional persistence (e.g. [1], [2]). The Critical Community Size (CCS) is the threshold population size below which an infectious disease is liable to stochastic extinction during post-epidemic troughs because the susceptible pool is exhausted [3], [4]. The larger the CCS, the greater will be the importance of rescue effects in maintaining circulation. Mass vaccination generally increases the probability of local extinction, potentially rendering rescue effects increasingly important as elimination thresholds are approached.

The CCS is a complex emergent function of pathogen biology and susceptible recruitment. Low birth rates (or R_0_, which has an analogous effect [5]), and high vaccine coverage increase the CCS; and, comparing infections, so does decreasing the infectious period [6], [7]. However, the relationship between R_0_ and the CCS may be complex. Although increasing R_0_ and birth rate tend in general to decrease the CCS (by lowering the susceptible threshold for epidemic growth), increasing mean transmission rate in the presence of seasonal forcing can decrease local persistence, as dynamics are drawn to multi-annual attractors, with deep inter-epidemic troughs [8]–[10].

Empirically, the CCS is normally estimated via the relationship between fadeouts (periods of zero incidence) and local community size [3], [10]–[12]. Underreporting of disease incidence may bias such estimates [13]. However, more importantly all local host communities are embedded in a metapopulation in which rescue effects will take place at least to some extent, and the rate and scaling of import of infected individuals change the nature of the relationship between zero-incidence and population size [9].

In an applied context, the CCS has been proposed as a guide for control strategies, and an argument has been made for ignoring communities below the CCS if vaccines are limited or resources constrained [14]–[16]. This, however, only has merit if regional persistence is largely due to local persistence in large (core) communities and if rescue effects are rare. The experience from attempting to eliminate vaccine preventable childhood infections shows that this is not always the case. For example, prior to recent outbreaks in Wales, contemporary measles in the UK persisted only through metapopulation processes [7], yet its country-wide persistence appears robust.

Despite its potential importance, the magnitude of rescue effects has not previously been empirically estimated. As local elimination, or even eradication targets are increasingly on the agenda, developing estimates of this effect is correspondingly important. Here we use national level data to explore this question globally. We use a comparison between island and mainland countries as a means to elucidate rescue effects, since greater requirements for reaching islands mean that they are likely to be less well connected to the global metapopulation. To further isolate this rescue effect, we control for covariates such as the size of the susceptible population (of critical importance in sustaining a chain of transmission), the human development index (likely to be indicative of the sensitivity of surveillance systems) and proportion of migrants living within the country (a measure of overall connectedness). We compare four immunizing infections with somewhat different life-histories.

## Methods

### Data sources

No community is completely isolated in this age of global travel. We therefore take a broad-brush global approach to study metapopulation persistence of rubella, measles, pertussis and mumps. We use yearly reported incidence available from the WHO [17]; and WHO corrected estimates of immunization coverage [18]. For rubella and mumps, vaccination coverage is not reported, so we used the WHO reported year of introduction for each country, and then assumed that thereafter, coverage reflects measles containing vaccine (MCV) coverage. For population size and birth rates, we use the World Bank data bank [19]. For estimates of the Human Development Index (HDI), we use UNDP data (http://hdr.undp.org/en/statistics/hdi/). To infer missing values of HDI, we used a polynomial regression linking log country GDP to available HDI values. The estimated relationship is *y* = 0.26–0.1731log(GDP)+0.0443log(GDP)^2^ –0.002 log(GDP)^3^, r^2^ = 0.88, p<0.05. We used the simplest possible classification for islands, including any country whose land mass was surrounded by water; with the exception of Australia, which is essentially a continent. Many resulting island countries are actually archipelagos (see Text S1 for the full list). To distinguish between highly connected islands (e.g., Singapore) and very remote islands (e.g., Vanu Atu) we also used a measure of the relative levels of movement between countries. Foreign-born and foreign-national population data derived from recent censuses represent the most complete and comparable datasets for global and regional analyses that most readily accord with actual population movements [20], [21]. We used these as a measure of the relative levels of movement between countries. Data on international bilateral migrant stocks for 226 countries and territories in 2000–2002 were obtained from World Bank estimates [20]. Wherever possible, these data were derived from the latest round of censuses, as these were considered most comparable at the global level. Where unavailable, population registers were drawn upon, and in the cases of missing data, a variety of techniques and tests were used to create and validate a complete matrix of international bilateral migrant stocks [21]. In total, all required data were available for between 12 (for rubella and mumps) and 30 (for pertussis and measles) years for 178 countries of which 40 were islands.

### Estimates of the Critical Community Size

To estimate the CCS, we took years for which incidence was reported (1998 to 2011 for rubella and mumps; and 1980 to 2011 for pertussis and measles), and identified the proportion of reported years with non-zero incidence. We then fitted a linear regression relating this to the size of the unvaccinated population, obtained by combining estimates of population size in every year for which data was available with estimates of vaccine coverage in that year, and then taking the average (resulting in one data-point per country). The CCS was crudely identified as the point at which this regression line intersects with zero. The covariate "unvaccinated population size" was chosen to facilitate comparison with previous analyses [10]–[12], despite the fact that unvaccinated births might be a correlate more tightly linked to persistence [8]. We also supply results using the latter instead, where unvaccinated births are obtained by multiplying births rates by population size to obtain the size of the birth cohort, and then multiplying this by 1-vaccination coverage to get the fraction unvaccinated. In fact, since infection for these diseases generally affects children at older ages, lagging this value might be appropriate; however, to avoid further depletion of the data, and given other likely greater imprecisions, we retained this value without further transformation.

### Estimating the Metapopulation Rescue Effect (MRE)

Many factors other than rescue effects affect the persistence of our four infections. We therefore use a logistic regression framework to study the probability of the chain of transmission being broken (at the annual time scale), as a function of country *i* being an island as well as a number of other potentially important covariates. Explicitly, we study the probability of zero incidence in year t+1 (*I_i,_*_t+1_ = 0) given pathogen circulation in the previous year (*I_i,_*_t_>0). We defined the indicator variable *J_i,_*_t+1_ = 0 when *I_i,_*_t+1_>0 and *J_i,_*_t+1_ = 1 when *I_i,_*_t+1_ = 0 and use this as the response variable. The covariates, other than whether the country was an island or not (*Isl*), are the log size of the population of unvaccinated children, *N*, taken to reflect susceptible population, the human development index, *HDI*, and the log of the proportion of the population made up of migrants living within the country, *M*. The model is of the form
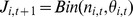



with, in the simple case of only main effects,
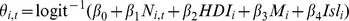



where *β_0_* is the intercept, *β_1_*–*β_4_* are slopes. We fitted all two-way interactions between the continuous covariates (*N*, *HDI* and *M)*, and identified the Minimum Adequate Model (MAM) using the ‘step’ function in R and then deleting interactions that were not significant at the 0.05 level (since we are interested in the island main effect, we did not include island interactions).

To carry out inference on the MRE that adequately incorporates parameter uncertainty, we used the variance-covariance matrix of parameters identified in the MAM, and generated 1000 samples from a multivariate normal with the appropriate mean and variance covariance [22]. This allowed us to obtain the odds of extinction on island countries relative to mainland countries incorporating parameter uncertainty. To extract the specific effect of islands, taken as representative of the MRE, we set other covariates to reflect an unvaccinated population size set at 100 000, the median HDI (0.67), and the median log proportion of migrants across countries (–3.52). Predictions can also be made that compare odds of extinction on an island with the lowest quantile of migrants (–6.25) vs. odds of extinction on the mainland with median (–3.25) or high quantiles of migrants (–0.62). However, since the number of migrants living within a country might be shaped by a range of historical, economical, and geographical factors, not necessarily of direct relevance to the metapopulation rescue effects, we present here results based on the median. The alternative analysis incorporating variation in number of migrants, yield the same qualitative predictions, but the estimated MREs are more extreme (Metcalf unpublished results).

## Results

**Figure 1:**
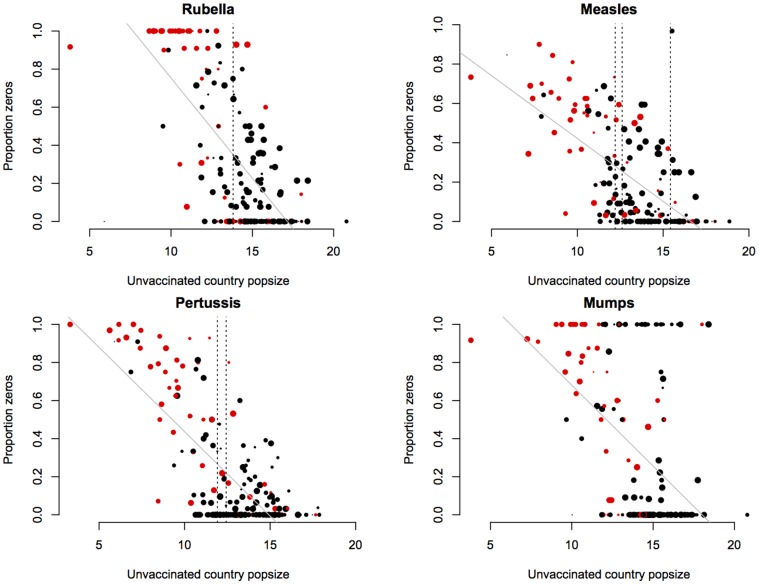
Critical Community Size of four immunizing childhood infections. The *x* axis shows the size of unvaccinated proportion of the populations of each country (log scale), the y axis shows the proportion of years where incidence reported to the WHO is greater than zero (years range between 1998 and 2011 for rubella and mumps and 1980 and 2011 for pertussis and measles). Colours indicate island states (red); the size of points indicates the number of years for which there was data. Vertical lines show previously reported CCS values; higher values for measles refers to Niger [10], lower to America and the UK [3], [24]; for pertussis values refer to England and Wales [12]. The grey lines show a fitted linear regression, weighted to reflect sample size for each country (Table 1). The extreme positive outlier for measles (reflecting no years with more than one case at a relatively large population size) is the Democratic People's Republic of Korea. For all infections, islands with no years with no cases reported tend to be islands like the United Kingdom and New Zealand, likely to have highly effective surveillance systems.

**Figure 2:**
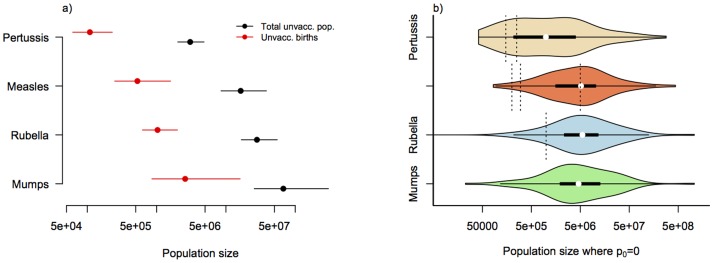
Estimates of the Critical Community Size. a) Estimates of the distribution of the population size at which no years with no cases are expected based on linear regressions described in Table 1 and shown in Fig. 1 for the total unvaccinated population and for unvaccinated births; here encompassing parameter uncertainty; b) Violin plots showing the distribution of sizes of the unvaccinated populations for which no years with no cases were recorded for each of the infections (p_0_ = 0 indicates no zeros in the time-series, and therefore points along the y = 0 line in Fig. 1); vertical dotted lines indicated previous estimates of the CCS for each of the infections, see Fig. 1 for a description.

The pattern of persistence against log unvaccinated population size is shown in Figure 1. Table 1 contains fitted regressions for both this relationship and the alternative obtained using log number of unvaccinated births as the covariate. Corresponding estimates of the CCS are shown in Figure 2a. The data show considerable spread along the x axis (Figure 2b), indicating that our CCS estimates carries substantial uncertainty. The ranges, however, encompass previous estimates for each of the three diseases for which estimates are available (Fig. 1 and 2). Despite our expectations, the proportion of the variance explained was higher for models using unvaccinated population size as a covariate, rather than models using the size of the unvaccinated birth cohort (Table 1, r^2^ values). There are two possible explanations. First, since the estimate of the size of the unvaccinated birth cohort combines three sources of information (population size, birth rate, and vaccination coverage) each with their own degree of uncertainty, the accumulated noise may reduce the signal. Alternatively, as the average age of infection for all infections considered is rather greater than 1, unvaccinated birth cohort in that year may not be the appropriate measure, and should instead be lagged to a degree reflective of the average age of infection. Since this may be a rather variable number even within countries [11] and given the limited number of years available for analysis, such lagging (by e.g., 9 years for mumps) would considerably deplete the number of data-points, we did not pursue this.

**Table 1:** Weighted linear regression linking log size of the unvaccinated population and proportion of years for which no cases were reported; followed by the same but taking the size of the number of unvaccinated births as the covariate; corresponding estimates of the CCS, obtained as the point at which the fitted line intersects with zero are shown in Fig. 2.

	Relationship to log size of the unvaccinated population		Relationship to the log number of unvaccinated births	
Rubella	y = 1.806–0.105x	r^2^ = 0.47, df = 187, p<0.05	y = 1.482–0.107x	r^2^ = 0.43, df = 181, p<0.05
Measles	y = 1.060–0.063x	r^2^ = 0.39, df = 187, p<0.05	y = 0.770–0.058x	r^2^ = 0.34, df = 186, p<0.05
Pertussis	y = 1.322–0.088x	r^2^ = 0.54, df = 187, p<0.05	y = 0.868–0.074x	r^2^ = 0.41, df = 186, p<0.05
Mumps	y = 1.152–0.084x	r^2^ = 0.24, df = 169, p<0.05	y = 1.227–0.083x	r^2^ = 0.19, df = 163, p<0.05

For all four infections, the data suggest lower persistence on islands. Parameter estimates for the minimum adequate model describing probability of extinction are shown in Table 2. All main factors are broadly consistent with theoretical expectations for persistence in epidemic metapopulations. The size of the unvaccinated child cohort has a negative effect on extinction probability. More unvaccinated children imply a greater susceptible replenishment rate and thus a more robust chain of transmission. There is a negative slope with HDI, consistent with the expectation that higher HDI implies greater health-care functioning (though any associated increase in reporting rates would be a confounder that pulls in the opposite direction). The log proportion of migrants in the population is expected to have a positive influence on persistence, consistent with more migration leading to more frequent rescue effects. This is clearly born out for measles and pertussis, and in a weaker and non-significant way for rubella and mumps. There is a significant interaction between HDI and size of the population of unvaccinated children for measles, indicating that larger populations with a high HDI more effectively control infections; and a significant interaction between the HDI and number of migrants for rubella, suggesting that more migrants have less of an effect in countries with a high HDI. In all cases, the direction of the effect of the "island" covariate was positive, indicating that persistence is lower (extinction rates higher) on islands (Table 2). The odds ratio of >1 indicate that the odds of extinction are greater on islands than on mainland countries. More specifically the odds of extinction on islands are roughly 1.5 fold higher on islands than on the mainland countries for measles; and are even higher for rubella, mumps and pertussis.

**Table 2:** Main effects for the model of the probability of extinction for each of the 4 infections identified using the ‘step’ function in R; and then eliminating variables not significant at the 0.05 level; standard errors shown in brackets; stars indicate significance with ° for p-values <0.1, * for p<0.05, ** for p<0.01, *** for p<0.001.

Description		Pertussis	Measles	Rubella	Mumps
**Intercept**	*β_0_*	4.79(0.71)***	7.09(1.77) ***	3.48(1.92) °	–8.01(5.40)
**slope of log unvaccinated children (** ***N*** **)**	*β_1_*	–0.60(0.05) ***	–1.08(0.17) ***	–0.28(0.06)***	–0.77(0.53)
		*OR: 0.548*	*OR: 0.340*	*OR: 0.755*	*OR: 0.46*
**slope of HDI (** ***HDI*** **)**	*β_2_*	–5.33(0.59) ***	–9.43(2.45) ***	–5.71(2.48) *	–8.41(7.26)
		*OR: 0.004*	*OR: 8.02e^–5^*	*OR: 0.003*	*OR: 0.0002*
**slope of log proportion of migrants (** ***M*** **)**	*β_3_*	–0.20(0.06) **	–0.30(0.05) ***	0.77(0.44)°	–0.23(0.174)
		*OR: 0.818*	*OR: 0.740*	*OR: 2.15*	*OR: 0.794*
**slope of island (** ***Isl*** **)**	*β_4_*	0.82(0.18) ***OR: 2.270	0.41(0.17) *OR: 1.506	0.68(0.31) *OR: 1.97	2.38(0.46) ***OR: 10.804

Residual deviances are 1140.4, 1318.3, 541.0, and 208.1 respectively in the order of the columns of the table; and degrees of freedom are 3513, 4004, 1044 and 203. Significant interactions were retained in the models, including an interaction between *N* and *HDI* for measles [0.85(0.25)***] and mumps [–1.626(0.779)*]; and an interaction between *HDI* and *M* for rubella [–1.39(0.62)*]. “OR” indicates the odds ratios corresponding to each covariate.

**Figure 3:**
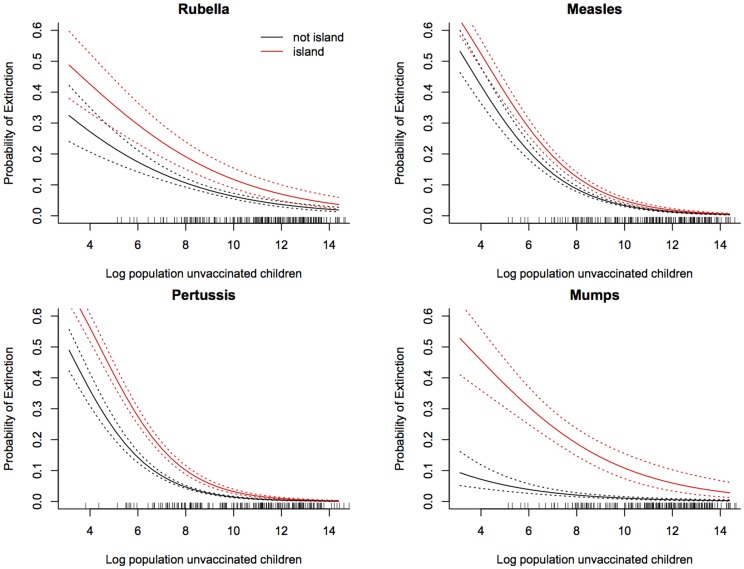
Predicted probability of extinction. The x axis is population size for four childhood infections and the y axis is probability of extinction for island nations (red) and mainland nations (black) and showing upper and lower standard errors, taken at the median log proportion of resident migrants (an index of connectivity of –3.52) and median human development index (0.67). Parameters underlying these predictions are shown in Table 1.

**Figure 4:**
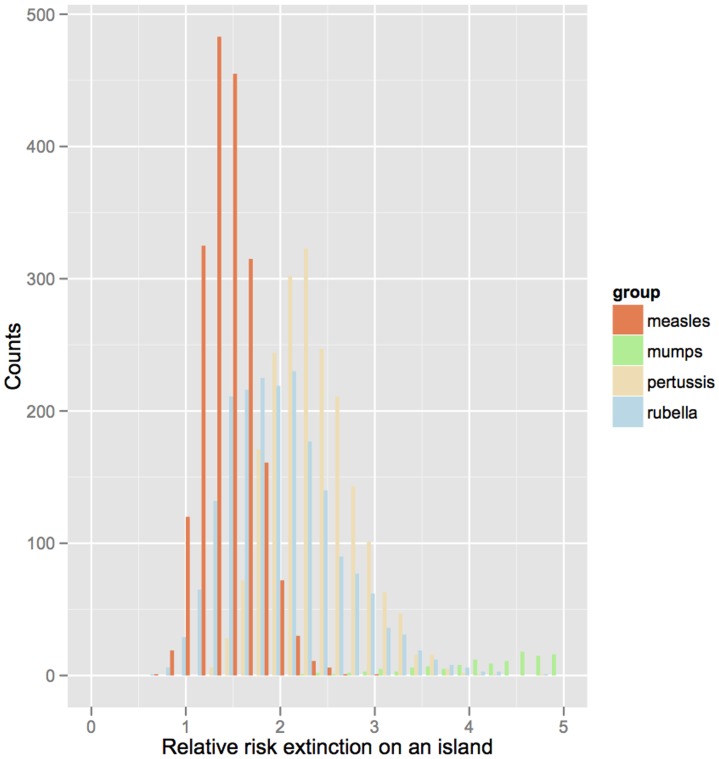
Relative risks of extinction on an island. The probability of extinction for island countries divided by the probability of extinction for non-island countries for 4 childhood infections relative to extinction on the mainland; showing only a fraction of the distribution for mumps for clarity. The proportion of migrants and HDI are set to the median across all countries (–3.52 and 0.67 respectively), and the population size of unvaccinated children is set to 1e5. Median values are 1.92 (1.04–3.37) for rubella, 1.51 (1.07–2.16) for measles, 10.26 (4.06–25.29) for mumps, and 2.27 (1.57,3.32) for pertussis; brackets indicate 2.5% and 97.5% quantiles from prediction made across 2000 samples from the estimated multivariate normal distribution of the parameters.

Correcting for all other covariates, Figure 3 depicts the probability of extinction as a function of population size on island *versus* non-island communities. To quantify the rescue effect, we further use the relative risk of extinction on islands (correcting for all other covariates and using resampling to propagate regression uncertainties) for the four infections (Figure 4). For all infections, the relative risks of extinction are predicted to be considerably higher in island than mainland countries (the bulk of the distributions shown in Fig. 4 are greater than 1). Note, though, that while there is a significant effect for all infections there is substantial uncertainty, once we propagate the regression errors. The extreme value estimated for mumps may be a real biological effect, or reflect biases because of the generally very low reporting rate for this infection. The measures of development (HDI) and migration had a lesser, yet still significant effect on the probability of disease extinction. For countries with high levels of development (HDI∼0.9), the probability of extinction tended to be around 1% higher than for countries with very low HDI (HDI∼0.4), whilst the probability of extinction decreased roughly in proportion with the percentage of migrants in the population.

## Discussion

Our analysis reveals a consistently higher risk of extinction of four different immunizing childhood infections on islands, when correcting for other confounding variables. We believe our study represents the first empirical estimate of the magnitude of metapopulation rescue effects (MREs) in epidemic metapopulations [2]. All else being equal, island nations have roughly double the risk of extinction for our four focal diseases (Figure 4), arguing for a key role for MREs in their persistence.

At the country scale, development level (as captured by the HDI) reduces infection persistence, and migration (measured by proportion of resident migrants) increases it. Both these aspects are likely to change dynamically across the globe in coming years, with global mobility and connectivity continuing to increase. Whether the combined impact of their future trajectories increases or decreases infection persistence within the global metapopulation will depend on the relative patterns of change and is an interesting question for future research.

A natural hypothesis for the ordering of the different infections in terms of odds of extinction on islands is that infections with longer generation times might be more sensitive to the MRE, and this is indeed what we observed, with a greater difference in extinction probabilities for pertussis and rubella on islands relative to measles. While mumps has a relatively long infectious period, it is an outlier in that the estimated MRE is very large. This may be a real effect or perhaps spurious because of the overall low reporting rates for mumps (estimated for example at 12% in pre-vaccination Copenhagen relative to 40% for measles and 17% for pertussis [23]).

Our analysis is subject to a number of uncertainties associated with the data used. These will variously pull the CCS to be either an over- or under-estimate. Widespread under-reporting of infections [10], [24], [25] should lead to an over-estimate of extinction risk, especially in countries where surveillance systems are weak. Additionally, countries with better vaccination coverage may also tend to have better surveillance, meaning that many countries with large unvaccinated birth cohorts will report too frequent absence of disease. Conversely, the coarseness of the annual time step will induce biases in the opposite direction, with short-lived fade-outs going undetected. Moreover the coarseness of the country-wide spatial grain will make more local fade-outs go undetected. Our estimates of the CCS (Figure 1) are generally slightly larger than those found previously [3], [10], [12], [24], implying that the crudeness of the spatial and temporal scale is counter-acted by biases acting in the other direction.

Despite the crudeness of our CCS estimates, we believe our estimates of the MREs to be more robust because they correct for a number of potentially confounding factors. It is further reassuring that the magnitude of the MREs are fairly consistent across the four diseases (except, perhaps, mumps), despite reporting rates being likely to differ. The variation in MREs correlates positively with infectious period and (possibly) negatively with disease severity. However, both these effects may be confounded by reporting rates, which are generally highest for measles (relatively virulent) and lowest for mumps (relatively avirulent). Furthermore, the more subtle effects of development (and therefore the effectiveness of health services) and population connectivity were still clearly apparent in the risk of disease extinction (Table 2).

In conclusion, we provide the first estimates of the magnitude of metapopulation rescue effects for four immunizing childhood infections, with values indicating an approximate halving of persistence in isolated nations. This is likely to be a highly significant effect from an applied point of view as mass vaccination increasingly drives persistence away from the local scale towards the metapopulation scale for such diseases. There is some indication that infections with longer generation times have stronger rescue effects, as consistent with metapopulation theory (Figure 4). Disease severity may also directly affect metapopulation rescue effects if illness decreases movement of infectious individuals, and likelihood of contact between susceptible and infectious individuals, although associated differences in reporting rate may confound such effects. The structure of within-country metapopulations will be a key determinant of the global patterns that we report; and will be a function of population density and distribution with countries, as well as area, and other geographic features (e.g., for island countries, the role of being an archipelago). More detailed information at finer spatial scales is increasingly available for specific countries [10], [11]; and comparative analyses of commonalities and determinants of countries’ metapopulation structures are increasingly possible, opening the way to generalization of the role of metapopulation rescue effects at a global scale.

## Supporting Information

Text S1 **List of Island Nation States considered.**
(DOC)
