# Supplementary material for: Persistence in Epidemic Metapopulations: Quantifying the Rescue Effects for Measles, Mumps, Rubella and Whooping Cough
Source: PLoS One. 2013 Sep 9;8(9):e74696. doi: 10.1371/journal.pone.0074696 (PMC3767637; doi:10.1371/journal.pone.0074696)
Supplement: Text S1 — List of Island Nation States considered. (DOC) [file pone.0074696.s001.doc]

**Supporting Information**: List of Island Nation States considered:

Antigua and Barbuda, Bahamas, Bahrain, Barbados, Brunei Darussalam, Cape Verde, Comoros, Cook Islands, Cuba, Cyprus, Dominica, Fiji, Grenada, Haiti, Iceland, Indonesia, Ireland, Jamaica, Japan, Kiribati, Madagascar, Maldives, Malta, Marshall Islands, Mauritius, Micronesia (Federated States of,) Nauru, New Zealand, Niue, Palau, Papua New Guinea, Philippines, Saint Kitts and Nevis, Saint Lucia, Saint Vincent and the Grenadines, Samoa, Sao Tome and Principe, Seychelles, Singapore, Solomon Islands, Sri Lanka, Timor-Leste, Tonga, Trinidad and Tobago, Tuvalu, United Kingdom, Vanuatu.
